# α-Actinin-4 Is Essential for Maintaining the Spreading, Motility and Contractility of Fibroblasts

**DOI:** 10.1371/journal.pone.0013921

**Published:** 2010-11-11

**Authors:** Hanshuang Shao, James H.-C. Wang, Martin R. Pollak, Alan Wells

**Affiliations:** 1 Department of Pathology, University of Pittsburgh, Pittsburgh, Pennsylvania, United States of America; 2 Department of Orthopaedic Surgery, University of Pittsburgh, Pittsburgh, Pennsylvania, United States of America; 3 Division of Nephrology, Department of Medicine, Beth Israel Deaconess Medical Center and Harvard Medical School, Boston, Massachusetts, United States of America; 4 Pittsburgh VA Health System, Pittsburgh, Pennsylvania, United States of America; University of Birmingham, United Kingdom

## Abstract

**Background:**

α-Actinins cross-link actin filaments, with this cross-linking activity regulating the formation of focal adhesions, intracellular tension, and cell migration. Most non-muscle cells such as fibroblasts express two isoforms, α-actinin-1 (ACTN1) and α-actinin-4 (ACTN4). The high homology between these two isoforms would suggest redundancy of their function, but recent studies have suggested different regulatory roles. Interestingly, ACTN4 is phosphorylated upon growth factor stimulation, and this loosens its interaction with actin.

**Methodology/Principal Findings:**

Using molecular, biochemical and cellular techniques, we probed the cellular functions of ACTN4 in fibroblasts. Knockdown of ACTN4 expression in murine lung fibroblasts significantly impaired cell migration, spreading, adhesion, and proliferation. Surprisingly, knockdown of ACTN4 enhanced cellular compaction and contraction force, and increased cellular and nuclear cross-sectional area. These results, except the increased contractility, are consistent with a putative role of ACTN4 in cytokinesis. For the transcellular tension, knockdown of ACTN4 significantly increased the expression of myosin light chain 2, a element of the contractility machinery. Re-expression of wild type human ACTN4 in ACTN4 knockdown murine lung fibroblasts reverted cell spreading, cellular and nuclear cross-sectional area, and contractility back towards baseline, demonstrating that the defect was due to absence of ACTN4.

**Significance:**

These results suggest that ACTN4 is essential for maintaining normal spreading, motility, cellular and nuclear cross-sectional area, and contractility of murine lung fibroblasts by maintaining the balance between transcellular contractility and cell-substratum adhesion.

## Introduction

α-Actinins, highly related members of the spectrin superfamily, are ubiquitously expressed. These proteins were originally described as the main actin-crosslinking proteins [1]. To-date, four isoforms have been identified. α-Actinin-2 and -3 are mostly expressed in muscle cells, whereas α-actinin-1 and -4 are present in non-muscle cells [2]. α-Actinins exist as paired head-to-tail homodimers that link to actin filaments through their N-terminal actin binding domains [3]. The c-terminal of α-actinins ends with EF hand motifs that have been shown to bind calcium. The central rod domain, composed of four spectrin-like repeats forming the antiparallel pairing, is thought to contribute to protein stability and ability to provide a mechanically elastic platform for the docking of other proteins [1].

Among these four actinin isoforms, α-actinin-4 (ACTN4), first cloned by Yamada's group [4], has been shown to play a crucial role in cancer invasion and metastasis [2], [4], [5], [6], [7], [8] and in maintaining normal kidney functions [9]. Mice deficient in α-actinin-4 present severe glomerular disease due to abnormal podocyte adhesion [9], [10]. The roles in cancer related to cell dissemination. Increased levels of α-actinin-4 enhance the motility of colorectal cancer cells [5] and down regulation of α-actinin-4 dramatically reduces the migration of glioblastoma cells [8]. The molecular basis of the above relates to the role of α-actinin-4 in crosslinking the cytoskeleton to focal adhesion and in the regulation of actin cortical network, as noted most dramatically during cytokinesis [11], [12]. Recently a novel molecular function has been proposed as α-actinin-4 has been shown to shuttle between cytoplasm and nucleus as it interacts with transcriptional regulators [13].

The functioning of α-actinin-4 is regulated by external signals. Upon the stimulation of cells with epidermal growth factor, tyrosyl-phosphorylation of α-actinin-4 impairs its actin binding activity allowing for dissolution of cytoskeletal bundles, a process required for both rapid cell migration and appropriate cytokinesis [12]. Although α-actinin-1 and α-actinin-4 have 87% identity in amino acids, only α-actinin-4 is enriched at the leading edges of invading cells [4], and the presence of α-actinin-1 did not blunt the cell behavioral alterations of α-actinin-4 knockdown [12].

Despite the accumulating evidence of increased α-actinin-4 levels as a biomarker for cancer invasion and metastasis, the actual cellular functions of ACTN4 remain undetermined. This is due, at least in part, to the numerous functioning signaling networks extant in cancer cells and tumors. In this study, we determined the cellular function of ACTN4 in a cell system relatively devoid of the autocrine signaling cascades. We queried murine lung fibroblasts in which ACTN4 has been stably and significantly downregulated [9]. We found that knockdown of ACTN4 significantly reduced cell motility, focal adhesions, and cell proliferation, but surprisingly increased the rate of cell spreading, and resulted in increased cell and nuclear cross-sectional area. This co-existed with enhanced transcellular contractility, potentially tipping the balance between adhesion and contractility. These data provide insights into the physiological and pathological roles of ACTN4 functioning.

## Materials and Methods

### Antibodies and cell lines

Antibodies against actinin-4 (catalog #: sc-49333), actinin-1 (catalog #: sc-135819), pan actinin (catalog #: sc-15335), non-muscle myosin heavy chain 9 (MYH9) (catalog #: sc-98978), non-muscle myosin heavy chain 10 (MYH10) (catalog #: sc-99210), and myosin II (catalog #: sc-53092) were purchased from Santa Cruz (Santa Cruz, CA). Polyclonal MLC2 antibody (catalog #: 3672) was purchased from Cell Signaling (Danvers, MA). Monoclonal vinculin antibody (catalog #: V9131) and polyclonal actin antibody (catalog #: A2668) were purchased from Sigma (St. Louis, MO). Wild type murine lung fibroblasts (WT ACTN4) and stable ACTN4 knockdown (ACTN4 KD) murine lung fibroblasts were provided by Dr. Pollak's laboratory. Cells were cultured for less than 13 passages. The cell lines were isolated from lungs of ACTN4^+/+^ and ACTN4^−/−^ mice [14]. In brief, lungs from neonatal ACTN4 mutant and wild type littermate mice were digested for 45 min at 37°C in RPMI with 0.28 U/ml liberase blendzyme 3 and 60 U/ml DNase I, passed through a 70 µm filter, centrifuged at 540 x *g* at 4°C, and plated in tissue culture flasks in DMEM with 15% fetal bovine serum (FBS). Cells were passaged when subconfluent after harvest with trypsin-EDTA. Both WT ACTN4 and ACTN4 KD fibroblasts were grown in DMEM (Cellgro, Lawrence, KS) with 10% FBS, 1× non-essential amino acids, 1× sodium pyruvate, 2 mM L-glutamine and 1× streptomycin/penicillin.

### Motility assay

Cell migration assessment was based on a motility assay described previously [15]. Briefly, cells of WT ACTN4 and ACTN4 KD murine lung fibroblasts were plated on 6-well plastic dishes and grown to confluence in DMEM in the presence 10% FBS. After overnight incubation, a scraped area was made by a rubber policeman. Over the next 24 hrs cells were allowed to migrate into this denuded area. Photographs were taken at 0 hr and 24 hr, and the relative distance migrated by the cells was determined by Photoshop software.

### Cell spreading assay

Both WT ACTN4 and ACTN4 KD fibroblasts were transiently transfected with eGFP and ACTN4 KD fibroblasts were also transiently transfected with human wild type ACTN4 or ACTN1 tagged with eGFP [12] using the lipofectamine 2000 reagent (Invitrogen, Carlsbad, CA) according to manufacturer's instruction in DMEM without fetal bovine serum. Six hours after transfection medium containing transfection reagent and plasmid was replaced with normal growth medium and further incubated for 16 hr. Fibroblasts were trypsined and plated on 6-well plastic dishes pre-coated with 1 µg/ml fibronectin and incubated at 37°C in 5% CO_2_ incubator for designated time periods. Cells were washed once with PBS and then fixed with 2% formaldehyde for 30 min at room temperature. After washing, images of WT ACTN4 expressing eGFP and ACTN4 KD expressing eGFP and ACTN4 KD expressing human ACTN4-eGFP/ACTN1-eGFP were taken. The area of fibroblasts was calculated by using ImageJ software.

### Centrifugal assay for cell adhesion

WT ACTN4 and ACTN4 KD fibroblasts were plated on 12-well plastic dishes pre-coated with 1 µg/ml fibronectin and cultured at 37°C in 5% CO_2_ incubator for 1 hr. Plates were inserted into a special box and completely filled with culture medium. This was placed in a hanging-bucket face down and then spun at designated centrifugation forces for 5 min to detach cells. The adhesive cells were washed once by PBS, fixed by formaldehyde and then stained using 0.5% crystal violet for 10 min at room temperature. After extensive washing with PBS, the color was extracted with 2% SDS solution and then transferred to a 96-well plate. The absorbance at 550 nm was measured by using a 96-well plate reader.

### Measurement of cellular and nuclear cross-sectional area

Both WT ACTN4 and ACTN4 KD fibroblasts were transiently transfected with eGFP and ACTN4 KD fibroblasts were also transiently transfected with human wild type ACTN4 tagged with eGFP as above. The day after transfection, cells were incubated with Hoechst 33342 (Sigma, St. Louis, MO) at a final concentration 10 µg/ml at 37°C in 5% CO_2_ incubator for 10 min prior to fixation with 2% formaldehyde for 30 min at room temperature. After washing, microphotogrpahs were taken of phase and fluorescent images of WT ACTN4 and ACTN4 KD expressing either eGFP or human ACTN4-eGFP/ACTN1-eGFP. The areas of nuclei and cells were calculated by using ImageJ software.

### Contractility assay

Cell traction force microscopy (CTFM) was used to assess cell contractility by measuring cell traction forces [16]. In brief, thin polyacrylamide gels (5% acrylamide and 0.1% bis-acrylamide) containing 0.5 µm red fluorescent micro-beads (Molecular probes, Eugene OR) were prepared on optical glass-bottom 35 mm dishes treated with 0.1% sodium hydroxide, 3-aminopropyltrimethoxysilane and 0.5% glutaraldehyde. The gel surface cross-linked with sulfo-SANPAH (Pierce, Rockford, IL) with the aid of ultraviolet (UV) light was coated with collagen type I (10 µg/ml) (BD Biosciences, Bedford, MA). After extensive washing with PBS, 3,000 cells in 130 µl volume were placed on gel and incubated at 37°C in 5% CO_2_ incubator for 1 hr to allow cells to attach. Floating cells were aspirated and 1 ml complete medium was added for additional 5 hr incubation. Phase contrast images of individual isolated cells that spread well and fluorescent images of red beads in the very top layer of the gel were taken. After cells were detached from the gel surface by treatment with sodium hydroxide, the fluorescent images of red beads at the same location were taken again. The cell traction forces were then computed by using a MATLAB program comparing bead displacement as previously described [17].

### Cellular compaction assay

Cellular compaction assay was performed using a previously described method [18]. In brief, WT ACTN4 and ACTN4 KD murine lung fibroblasts were harvested from monolayer culture using 0.25% trypsin/EDTA and resuspended in growth media. Neutralized collagen solution (1 mg/ml) (BD Biosciences, Bedford MA) containing 10^6^ cells/ml was dispensed into 24-well culture plates (0.5 ml solution/well). Collagen solutions were left to polymerize for 60 min at 37°C in a humidified incubator with 5% CO_2_ after which each matrix was overlaid with 1 ml of growth media. The matrices were gently released from the surface and sides of each well using a scalpel and incubated for 24 hr. Compaction was determined by measuring the area of collagen gel by using PhotoShop software.

### RNA isolation and quantitative PCR

Total RNA was isolated from WT and KD fibroblasts using the Trizol Reagent (Invitrogen, Carlsbad, CA) according to manufacturer's instructions. cDNAs of both WT ACTN4 and ACTN4 KD were generated from 1.5 µg total RNA using Omniscript RT kit (Qiagen, Valencia, CA) following the manufacturer's instructions. Quantitative PCR was performed in 25 µl reaction volumes containing 1 µl of cDNA, 0.25 µM each of forward and reverse primer, and 12.5 µl of Stratagene Brilliant SYBR® Green QPCR Master Mix (Agilent, Santa Clara, CA). PCR Reaction conditions included a 10 min pre-denaturing step at 95°C, followed by 35 cycles of 30 seconds at 95°C, 30 seconds at 58°C, and 1 min at 72°C. The primers used for qPCR are: myosin IIA forward: 5′-gaagaaggagcaggacactag, reverse: 5′-cctttgacggcctcagcattc; myosin IIB forward: 5-ctgaagaaggagcaggacacc, reverse: 5-cccttgacggcttcgatgttg; MYH9 forward: 5-accggctgaagaaggccaacc, reverse: 5′-cagctgctcctccagctgtg; MYH10 forward: 5′-accgcttccgcaagaccacgc, reverse: 5′-aagctgctcctccagctgcc; MLC2 forward: 5′-agagaagggcaggagcggaag, reverse: 5′-tggcttccttcatcatagcgtc; MLC regulatory B peptide forward: 5′-gatttaaaccgccaccatgtc, reverse: 5′-gtccaggtaggcatcagtggg; GAPDH forward: 5′-gaagcaggcatctgagggcc, reverse: 5′-aggccatgtaggccatgagg. Quantitative PCR data were normalized to a standard curve. Data for target genes were then normalized to the expression levels of GAPDH. Finally, target genes of KD fibroblasts were normalized to those of WT.

### Proliferation assay

Cell proliferation was assessed by manually counting the number of viable cells. In brief, cells grown in 6-well plate were detached with trypsinization and then resuspended in appropriate volume of complete growth medium. Finally, cells incubated with 0.2% trypan blue for 5 min at room temperature were loaded on hemacytometer. All unstained cells residing in all areas were counted under light microscope and the total number of viable cells per well were correctly calculated.

## Results

### Knockdown of ACTN4 impairs migration of murine lung fibroblasts

**Figure 1:**
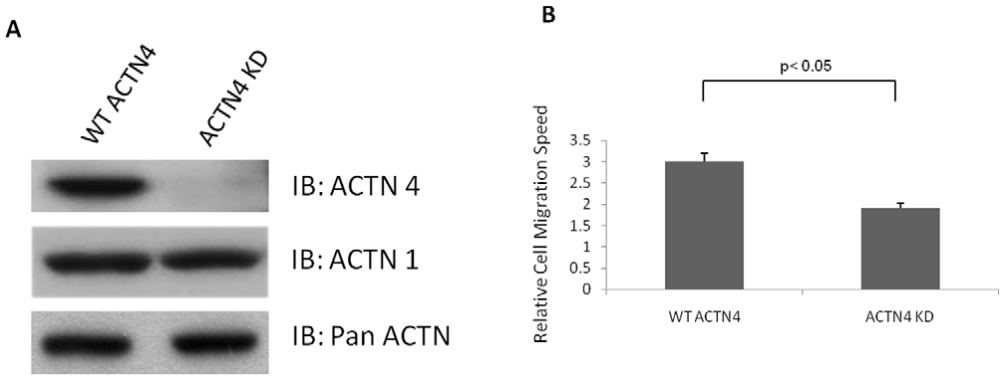
Knockdown of ACTN4 impairs cell migration of murine lung fibroblasts. (A) WT ACTN4 and ACTN4 KD murine lung fibroblasts grown in 6-well Petri-dish plates were lysed with RIPA buffer in the presence of 1× proteinase cocktail mix. Equal amount of total protein were separated by 7.5% SDS-PAGE and immunoblotted with anti-ACTN4, ACTN1 and pan-ACTN. The data are representative of three independent experiments. (B) Cells were plated on 6-well plastic dishes and grown to confluence in DMEM in the presence 10% fetal bovine serum. A scraped area was made by a rubber policeman and then cultured for additional 24 hr. Photographs were taken at 0 hr and 24 hr, and the relative distance migrated by the cells was determined by using Photoshop software. Data are mean ± s.e.m., measured from three independent experiments each in triplicate.

ACTN4 has been shown to be required for normal podocyte adhesion [10] and mice deficient in ACTN4 have severe glomerular disease presumably due to this altered adhesiveness [9]. In order to detect the cellular functions of ACTN4, we tested both wild type ACTN4 (WT ACTN4) and knockdown (ACTN4 KD) murine lung fibroblasts. We found that ACTN4 KD fibroblasts had no detectable ACTN4 protein compared to wild type cells, but ACTN1 and pan-ACTN (recognizing ACTN1, 2 and 3) detected proteins at similar levels in the two cell types (Fig. 1A). The KD fibroblasts were named as ACTN4^−/−^ in the original description [14] due to undetectable expression of ACTN4 in mice kidneys at both the protein and mRNA levels. As ACTN4 is differentially expressed in different organs [9], we re-examined this in fibroblasts. We detected trace levels of ACTN4 mRNA in ACTN4^−/−^ fibroblasts without evidence of protein. This is likely due to the original “knock-in” strategy not deleting ACTN coding sequences, but rather the construct appeared to alter transcription or transcript stability. Thus, we now refer to the “ACTN4^−/−^ fibroblasts” as knockdown (KD) instead of “knockout”, even though we do not detect protein in these cells.

Migration integrates many functions related to adhesion and cytoskeletal construction and control. Thus we determined whether ACTN4 is required for cell migration by performing an *in vitro* ‘wound healing’ assay. We found that knockdown of ACTN4 significantly impaired the motility of murine lung fibroblasts (Fig. 1B). This result suggests the ACTN4 is essential for maintaining normal cell motility, and thus we sought to examine the various cell biochemical aspects of migration.

### Knockdown of ACTN4 hastens cell spreading and changes cellular morphology

**Figure 2:**
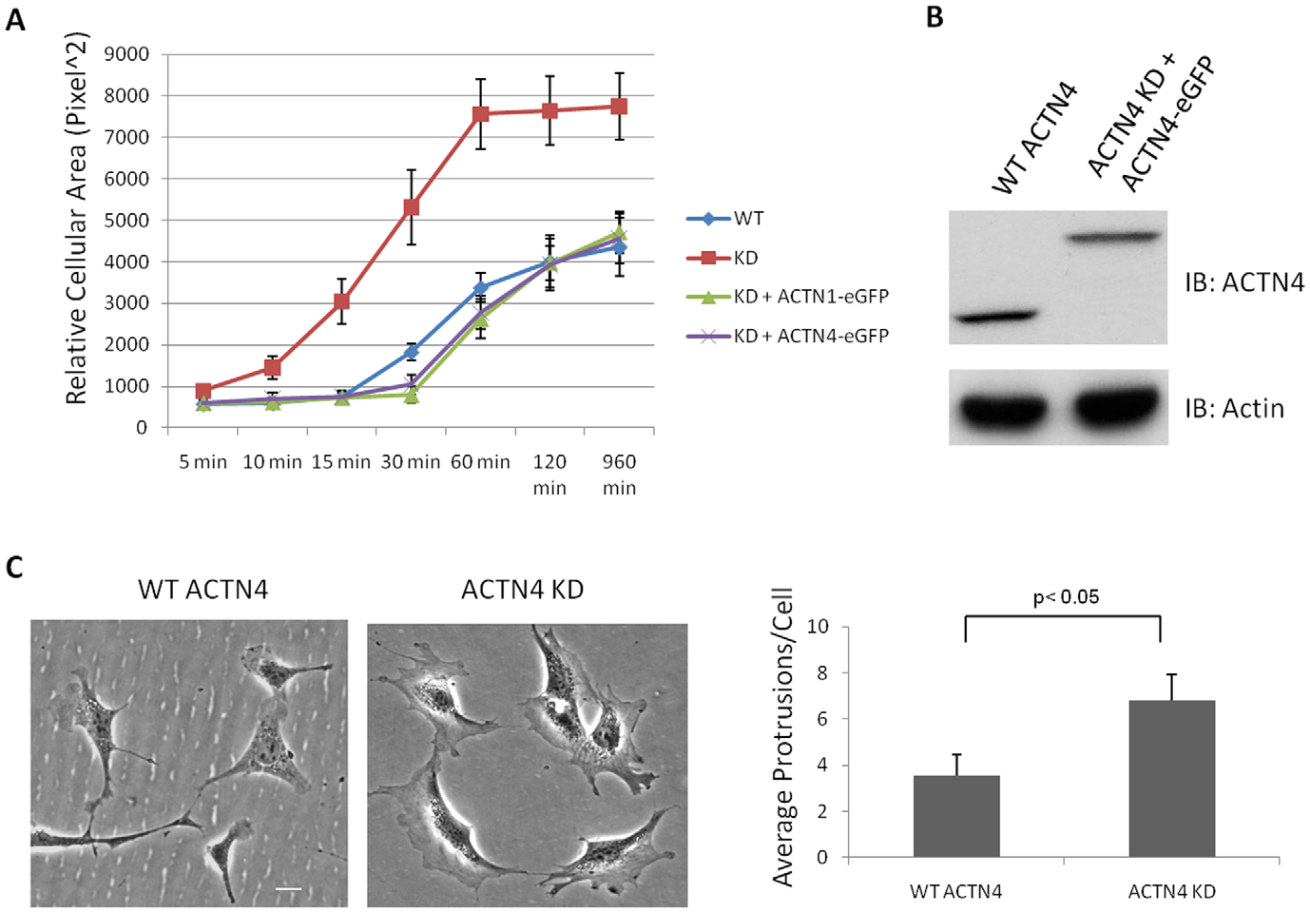
Knockdown of ACTN4 hastens cell spreading of murine lung fibroblasts. (A) Murine lung fibroblasts (WT ACTN4 and ACTN4 KD) were transiently transfected to express the designated constructs. These cells were trypsined and then plated in 6-well plates coated with 1µg/ml fibronectin. After culturing for 5 min, 10 min, 15 min, 30 min, 1 hr, 2 hr and 16 hr, respectively, unattached cells were washed away with PBS. Adhesive cells were fixed with 2% formaldehyde for 30 min at room temperature followed by imaging under fluorescent microscope. The cellular cross-sectional area of attached cells with GFP fluorescence was measured by using ImageJ software. Quantification represents the average cellular cross-sectional area (± the standard deviation) of at least 50 individual fluorescent cells chosen randomly. (B) Immunoblotting of endogenous ACTN4 and exogenous human wild type ACTN4-eGFP. The immunoblotting results were the representative of three independent experiments. (C) Morphology of both WT ACTN4 and ACTN4 KD fibroblasts grown in Petri dishes and the quantification of protrusions per cell. Images present both WT and KD fibroblasts chosen randomly. Quantification represents the average protrusions per cell (± the standard deviation) of at least 50 individual cells chosen randomly. Bar  = 5 µm.

The actin cytoskeleton plays a crucial role in cell spreading [19]. As ACTN4 is an actin cross-linking protein, we sought to determine the role of ACTN4 in cell spreading. To our surprise, in the absence of ACTN4, cells started to spread 5 min after seeding and almost reached the maximum cellular cross-sectional area within an hour. In contrast, WT ACTN4 cells had a 10 min delay in the start of spreading and then only slowly reached its maximum extent (requiring more than two hours) (Fig. 2A). In order to investigate if the faster cell spreading was specifically caused by knockdown of ACTN4 gene, we re-expressed human wild type ACTN4 tagged with enhanced green fluorescent protein (eGFP) in ACTN4 KD murine lung fibroblasts to restore cell spreading. As shown in Fig. 2A, re-expression of human wild type ACTN4 efficiently retarded the cell spreading, slowing it to kinetics similar to those of WT fibroblasts. The re-expression of human wild type ACTN4-eGFp in ACTN4 KD fibroblasts was confirmed by immunoblotting (Fig. 2B).

As ACTN1 and ACTN4 are 87% identical in their amino acid sequences, we tested if ACTN1 could replace ACTN4 and restore cell spreading of ACTN4 KD fibroblasts. By overexpressing human ACTN1-eGFP in ACTN4 KD cells, we found that overexpression of ACTN1 also efficiently restored cell spreading of ACTN4 KD cells (Fig. 2A). Possible reasons for this seemingly discrepant result [2] are discussed below.

Our previous study showed that human WT ACTN4-eGFP overexpressed in NR6WT fibroblasts accumulated in lamellipodia [12]. As shown in Fig. 2C, the downregulation of ACTN4 significantly changed the cellular morphology of ACTN4 KD murine lung fibroblast. Predominant lamellipodia were present in WT ACTN4 fibroblasts. In contrast, ACTN4 KD cells presented twice as many protrusions, without a main lamellipod, as the wild type cells. These results suggested that the ACTN4 is required for normal cell spreading and maintenance of cellular morphology.

### Knockdown of ACTN4 impairs focal adhesion strength

**Figure 3:**
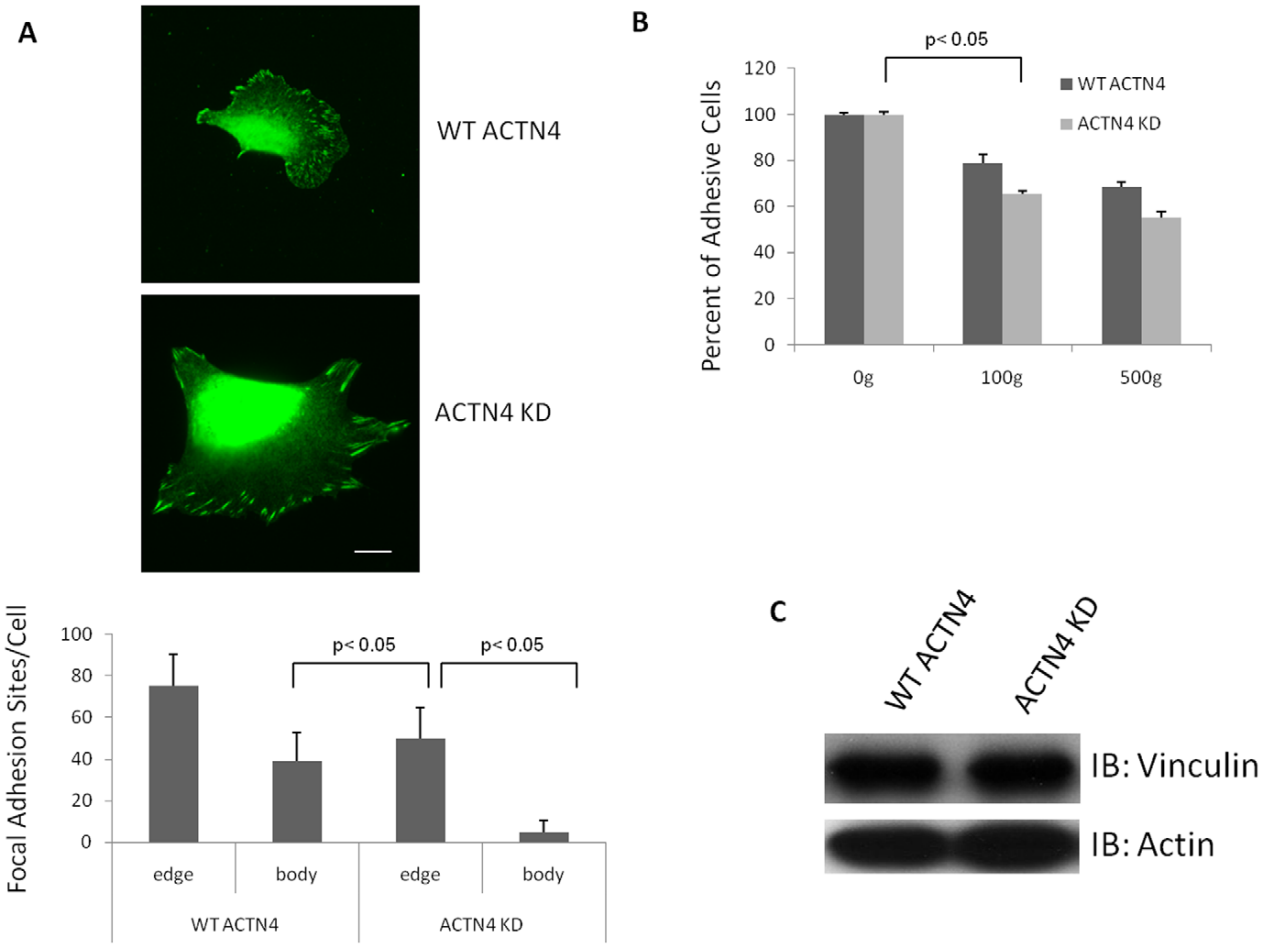
Knockdown of ACTN4 limits the number of focal adhesions. (A) WT ACTN4 and ACTN4 KD murine lung fibroblasts were stained for vinculin aggregates as indicative of focal adhesions. The number of focal adhesion sites residing within cell body and cellular edge were calculated by using ImageJ software for at least 30 individual cells. The ratio of focal adhesion sites between cell body and edge was also calculated. Images were taken randomly. Quantification was measured from at least 30 individual cells (± the standard deviation) chosen randomly. Shown are representative of at least independent experiments. Bar  = 10 µm. (B) An inverted centrifugation focal adhesion assay was performed (see “material and method” for details). Data are mean ± s.e.m., measured from three independent experiments. (C) Immunoblotting shows that the vinculin level was similar in WT and KD cells. Blot is representative of three independent assessments.

The above data showed that the knockdown of ACTN4 in lung fibroblasts led to faster cell spreading. Next, we investigated if the cell spreading of ACTN4 KD was related to alteration in the focal adhesions. We checked strength of adhesion using a centrifugal assay *in vitro* and adhesions numbers and structures by visualizing vinculin, a focal adhesion marker. Interestingly, the immunofluorescence of vinculin showed that the ACTN4 KD murine lung fibroblasts had large and long focal adhesion sites at the edge of cell and the cell body had a few focal adhesion sites (Fig. 3A). In contrast, the focal adhesion sites of WT ACTN4 fibroblasts were localized homogeneously under the whole cell. As shown in Figure 3B, knockdown of ACTN4 decreased the adhesiveness of the cells soon after plating on plastic surfaces coated with fibronectin. The decrease in focal adhesion formation of ACTN4 KD was not due to the alteration of the expression of vinculin (Fig. 3C). This data suggested that the ACTN4 is required in the establishment of strong cell focal adhesions.

### Lack of ACTN4 leads to increased cell and nuclear cross-sectional area

**Figure 4:**
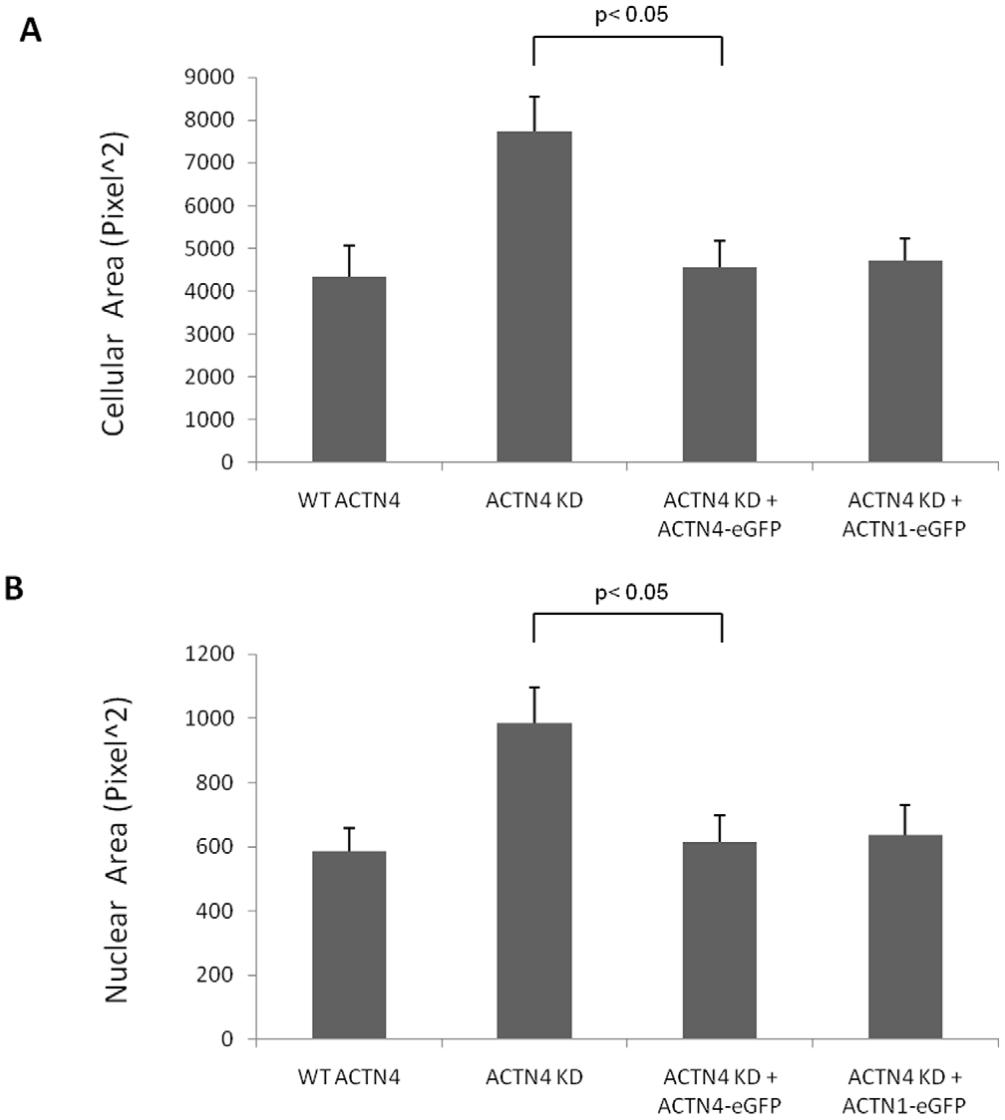
Knockdown of ACTN4 increases both cellular and nuclear cross-sectional area of murine lung fibroblasts. Murine lung fibroblasts of WT ACTN4 and ACTN4 KD transiently expressed eGFP and ACTN4 KD transiently expressed ACTN4-eGFP or ACTN1-eGFP were trypsined and then plated in 6-well Petri-dish plates coated with 1 µg/ml fibronectin. After incubation for 16 hr, cells were further incubated with 10 µg/ml Hoechst 33342 for 10 min at 37°C in 5% CO_2_ incubator prior to fixation with 2% formaldehyde for 30 min at room temperature. After taking fluorescent images under microscope, the cellular cross-sectional area of attached cells with GFP fluorescence (A) and the nuclear cross-sectional area with blue fluorescence (B) were measured by using ImageJ software. Quantification represents the average cellular and nuclear cross-sectional area (± the standard deviation) of at least 50 individual cells chosen randomly.

The foregoing highlighted cell spreading and extensions. As actin filaments and tubulins are involved in maintaining normal cellular and nuclear cross-sectional area, we sought to determine whether knockdown of ACTN4 affected the cellular and nuclear cross-sectional area of lung fibroblasts. As shown in Fig. 4A and 4B, knockdown of ACTN4 significantly increased the cellular and nuclear cross-sectional area. Re-expression of human ACTN4-eGFP or ACTN1-eGFP in ACTN4 KD fibroblasts almost completely restored both cell body and nucleus to normal area. This result strongly suggested that the ACTN4 is essential for maintaining normal cellular and nuclear area and that both ACTN1 and ACTN4 are able to contribute to the maintenance of cellular and nuclear area.

### Knockdown of ACTN4 increases cell traction force and cellular compaction

**Figure 5:**
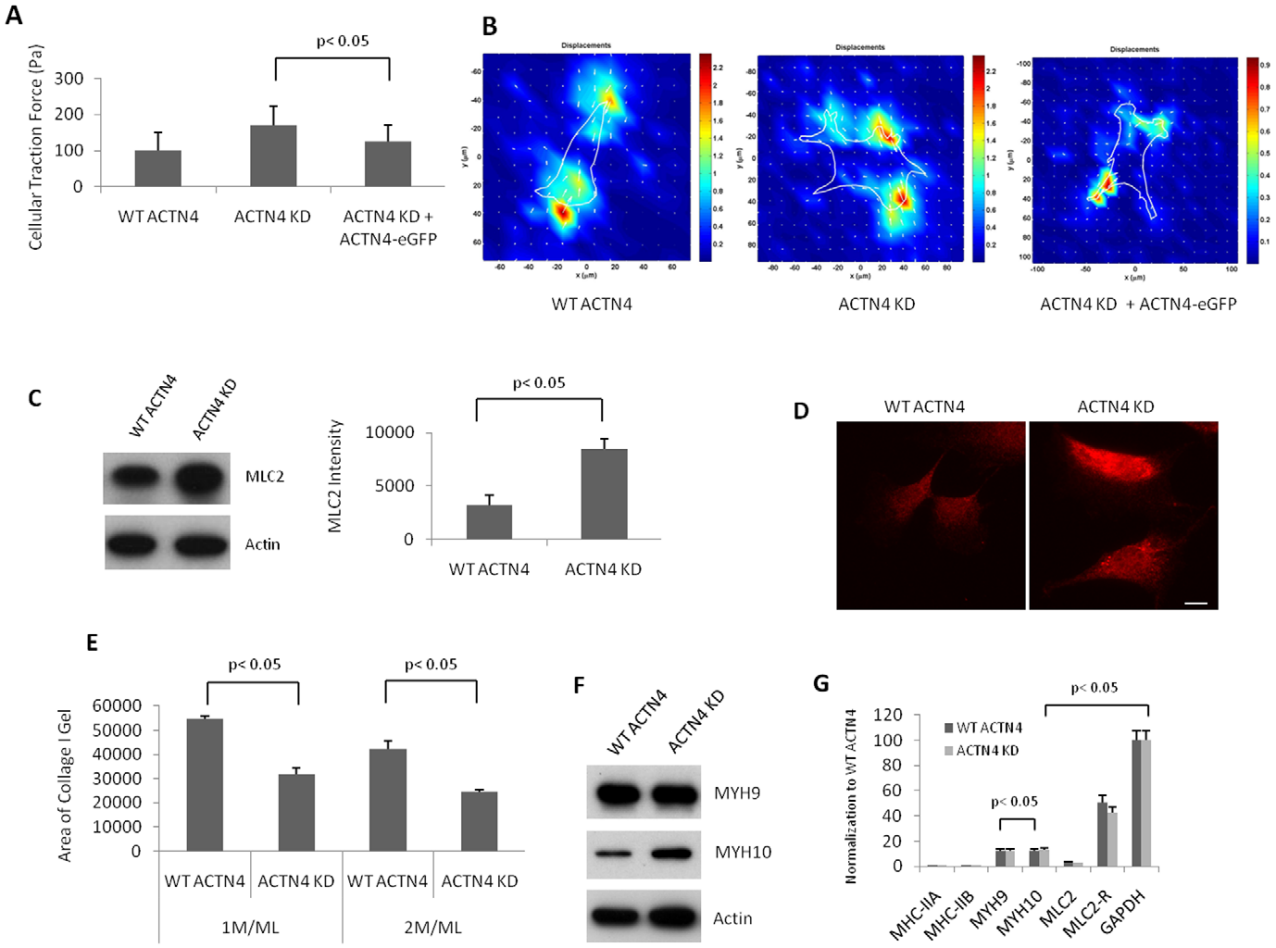
Knockdown of ACTN4 increases both contractile force and gel compaction generated by murine lung fibroblasts. (A) Contractile force assays were performed with both WT ACTN4 and ACTN4 KD murine lung fibroblasts transiently transfected with either eGFP or human wild type ACTN4-eGFP. For each cell type, at least 20 individual cells with GFP fluorescence were analyzed. Quantification represents the average traction force per cell (± the standard deviation) of at least 20 individual fluorescent cells chosen randomly. Shown are representative of at least independent experiments. (B) Images created by using special software. The length of arrows stands for the strength of traction force. Shown are representative images of three independent experiments. (C) Immunoblotting of MLC2 of total cellular lysate and actin as loading control. The bands of MLC2 were quantitated by using ImageJ software. Shown are representative blot of three independent experiments. Data are mean ±., measured from three independent experiments. (D) Immunofluorescence of MLC2 in both ACTN4 and ACTN4 KD murine lung fibroblasts. Images are representative of cells chosen randomly from three independent experiments. Quantification represents the average fluorescence (± the standard deviation) of at least 50 individual fluorescent cells chosen randomly. Bar  = 10 µm. (E) A compaction assay was performed with WT ACTN4 and ACTN4 KD fibroblasts. Data stands for the area of gel top surface after 24 hr incubation. Data are mean ±., measured from three independent experiments. (F) Immunoblottings of MYH9 and MYH10 of total cellular lysate and actin as loading control. Shown are representative blots of three independent experiments. (G) Quantitative PCR (see “methods and materials” for details). Data are mean ±. s.e.m., measured from three independent experiments each in triplicate.

During cell motility, cells need to generate transcellular contractile forces to retract the trailing uropodia. As actin microtubule cytoskeleton and myosin were involved in regulating the cell traction force, we hypothesized that knockdown of ACTN4 affected the contractility of fibroblasts. To test this hypothesis, we performed a cell traction florescent microscopy assay *in vitro* to measure the contractility of both WT ACTN4 and ACTN4 KD fibroblasts. As shown in Figure 5A, knockdown of ACTN4 significantly increased the cell traction force of fibroblasts. The traction forces in all cell types mainly localized at the edge of cell with the cell body exerting only very weak traction forces (Fig. 5B). This increase likely reflects the large but peripheral adhesions noted in Fig. 3A. To determine whether the increase of traction force was specifically caused by the lack of ACTN4, we re-expressed human ACTN4-eGFP in ACTN4 KD to restore the cell traction force. As shown in Fig. 5A &B, re-expression of human ACTN4-eGFP in ACTN4 KD fibroblasts almost completely reduced the cell traction force to the level of WT ACTN4 fibroblasts. This result strongly suggested that the ACTN4 is essential for maintaining normal cell traction force.

As recent reports showed that the reduced contractility of glioma in which ACTN4 has been significantly downregulated with siACTN4 RNA was due to the decreased expression of myosin II [8], we determined the expression of myosin II in both WT ACTN4 and ACTN4 KD murine lung fibroblasts. Immunoblotting results revealed that both WT ACTN4 and ACTN4 KD fibroblasts expressed very similar level of myosin II although their expression levels of both protein (data not shown) and mRNA were very low (Fig. 5G). Myosin II is composed of two heavy chains and four light chains. Myosin light chain 2 (MLC2), a regulatory subunit of myosin II has been shown to play an important role in the regulation of contractility and compaction of fibroblasts NR6WT [18]. Thus we next determined if the knockdown of ACTN4 affected the expression of MLC2 of murine lung fibroblasts. To our surprise, the expression of MLC2 is significantly increased in ACTN4 KD fibroblasts relative to WT ACTN4 fibroblasts by both immunoblotting (Fig. 5C) and immunofluorescence (Fig. 5D).

We queried the basis of changes in myosin-related proteins at the transcript level. As shown in Fig. 5G, knockdown of ACTN4 did not affect the mRNA level of myosin IIA, IIB and MLC2 although MLC regulatory B mRNA level was slightly downregulated in ACTN4 KD fibroblasts. Surprisingly, both WT and KD fibroblasts expressed both MYH9 and MYH10 at high levels with an increase of MYH10 but not MYH9 at the protein level in KD fibroblasts (Fig. 5F). However, the mechanisms by which ACTN4 knockdown enhances the expression of both MYH10 and MLC2 expression and slightly decreases the expression of myosin light chain regulatory B peptide in fibroblasts remain elusive and beyond the scope of the present manuscript.

To validate this force measurement, we analyzed the ability of cells to compact a matrix, which is a key function of fibroblasts during dermal regeneration [20], [21]. As shown in Fig. 5E, the compaction of floating collagen I gel was significantly greater in ACTN4 KD fibroblasts comparing to WT ACTN4. These results suggest that ACTN4 is required in regulating cell traction of fibroblasts and the increased contractile force and compaction was due to the whole cell changes, including any compensatory alterations in the proteome.

### Knockdown of ACTN4 decreases cell proliferation

**Figure 6:**
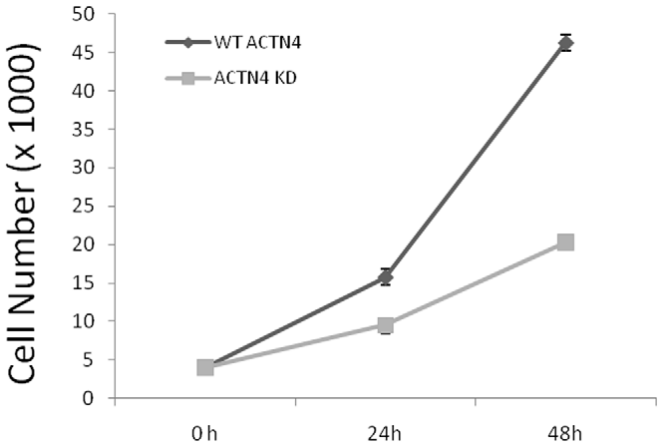
Knockdown of ACTN4 decreases cell proliferation. Both WT ACTN4 and ACTN4 KD fibroblasts were seeded at a density of 40K cells per well in 6-well Petri-dish plates. After culturing for appropriate time, all cells were harvested and only viable cells were counted on a hemacytometer. Data are the mean ± s.e.m., measured from three independent experiments each in triplicate.

Transcellular contractility and adhesion affect more than migration, as the same structures are not only required for permissive signals to drive proliferation [22], [23], but also regulate cytokinesis during mitosis. Additionally, previous reports [11], [12] have suggested a role for ACTN4 in maintaining the normal equatorial actin filaments during cell division. We sought to determine if the knockdown of ACTN4 affects cell proliferation. As shown in Figure 6, ACTN4 KD fibroblasts proliferated significantly slower compared to the WT ACTN4 fibroblasts, suggesting that ACTN4 is required for maintaining normal cell division. This finding was in line with the current report about the effect of ACTN4 on proliferation of astrocytoma cells [2].

## Discussion

Cell migration requires coordinated reorganization of cytoskeleton, lamellipodial extension, formation of forward adhesions, exertion of contractile force to pull the cell body forward, and the detachment of the rear [18], [24]. α-Actinins originally discovered as actin linking proteins have been shown to play a key role in regulating cell migration. In the past decades, accumulating evidences revealed that α-actinins interact with a large number of proteins including many transcription factors, in addition to actin [1], [13]. While both α-actinin-1 and α-actinin-4, two non-muscle isoforms have been shown to localize along the stress fiber, α-actinin-4 also accumulates at the leading edge of invading cancer cells [4] and motile fibroblasts [12]. This is consistent with α-actinin-4 being reported to play a crucial role in cancer invasion and metastasis [2], [4], [5], [6], [7], [8].

In this study, we found a counter-intuitive cellular function of α-actinin-4 in fibroblasts while dissecting its role in migration; we used a stable cell line of murine lung fibroblasts in which α-actinin-4 has been almost completely eliminated. In line with previous reports [2], [8], we found that knockdown of α-actinin-4 significantly impaired the cell motility of murine lung fibroblasts. Furthermore, our results showed that the impaired migration of fibroblasts was probably due to the scarcity of focal adhesion within cell body of ACTN4 KD fibroblasts and the elongation of focal adhesion sites at the periphery. The impaired focal adhesion of ACTN4 KD fibroblasts was not due to the alteration in level of the adhesion molecule vinculin (Fig. 3C). However, we could not directly attribute the migration to the number or location of adhesions. As adhesion assembly and turnover are highly dynamic and orchestrated processes essential for cell migration [22], we determined if the impaired cell migration of ACTN4 KD fibroblasts was due to the decreased turnover rate of focal adhesion at periphery. We found that the knockdown of ACTN4 did not affect turnover of focal adhesion (data not shown). However, the impaired cell migration of ACTN4 KD fibroblasts may also be due to the increased number of protrusions in ACTN4 KD fibroblasts as cells were impaired in their ability to establish a cell polarity for productive locomotion.

Recently, Choi and his colleagues have found that Myosin II is required for the elongation and maturation of adhesion [25]. Thus we sought to determine if the enlarged focal adhesion sites at the periphery of ACTN4 KD fibroblasts were caused by the increase of myosin II expression. As shown in Fig. 5G, our qPCR result showed that knockdown of ACTN4 did not affect the expression of myosin II in fibroblasts although the amount of myosin II mRNA was low. To our surprise, MYH9 and MYH10 in WT ACTN4 and ACTN4 KD fibroblasts are abundant and both MYH9 and MYH10 co-immunoprecipitate with an ACTN4 mutant (unpublished data). Knockdown of ACTN4 significantly increased the expression of MYH10 but not MYH9 at protein level instead of mRNA transcription (Fig. 5F and 5G). MYH9 and MYH10 probably substitute for myosin IIA and IIB, respectively. The increase of MYH10 expression in ACTN4 KD fibroblasts may cause the formation of enlarged focal adhesion sites at the periphery although the mechanism remains to be determined.

Recent reports suggested that the ACTN4 may play an important role in transcription regulation [13]. Indeed, current findings [8] and our results showed that the downregulation of ACTN4 significantly affected the expression of myosin II in Glioma and MYH10 and myosin light chain (MLC2) in fibroblasts. The increase of traction strength of the KD cells was likely due to either reduced actin filament bundling [26] or enhanced expression of MLC2, a contractile machinery element. The adhesive strength at one hour after plating was less in these cells (Fig 3B), but that is consistent with the increased traction strength as this would counter the ability to establish a large number of adherent sites shortly after attachment.

Our results showed the opposite cellular function of α-actinin-4 on focal adhesion and contractile force in fibroblasts compared to those reported in glioma cells suggesting that the integrated cellular functional consequences of α-actinin-4 activities are partially dictated by the cell type. Although the downregulation of ACTN4 caused a significantly increase in traction force of ACTN4 KD fibroblasts, these cells migrated slowly relative to WT ACTN4 fibroblasts. This was probably due to two reasons. First, the direction of all traction force faced towards the central cell body from the cellular periphery instead of facing one direction. Second, ACTN4 KD fibroblasts produced several “tail-like” structures but failed to produce a dominant protrusion (or cell polarity) although its focal adhesions within the cell body were impaired. In order to determine if the impaired migration of KD cells was partially due to the reduced proliferation (Fig. 6), we measured the motility of KD cells quiesced with quiescence medium in the absence of FBS for 24 hr by performing a standard scraped wound healing assay and found that quiesced KD cells still migrated significantly slower than WT cells (data not shown). This suggested that the impaired motility of KD cells was not caused by the reduced proliferation.

Our findings also suggested that the ACTN4 is essential for maintaining normal cellular and nuclear cross-sectional area. However, the mechanisms of how ACTN4 plays an important role in controlling cellular and nuclear cross-sectional area are still unclear. For the first time, our results also showed that the knockdown of α-actinin-4 also significantly impaired fibroblasts proliferation. This is not surprising as several reports in the literature revealed a function for α-actinin-4 in cytokinesis during mitosis [11], [12], [13], [27].

One question remains as to why there are multiple α-actinin isoforms. Although the identify of amino acids between α-actinin-1 and α-actinin-4 is 87%, these two isoforms are reported to play contrasting roles in cancer cell survival and the effect of ACTN4 shRNA on cell growth even depends on the cell type [2]. Still, in our hands, we could restore the normal cell phenotype by expressing ACTN1 in lieu of ACTN4, suggesting that these two proteins are potentially interchangeable. This is a conundrum in that in the absence of ACTN4, the endogenous ACTN1 did not prevent the phenotypic changes, nor was ACTN1 compensatorilly upregulated. Thus, either there is a protein dosage aspect that was restored by expression of either ACTN4 or ACTN1, or the forced expression of ACTN1 led to aberrant cellular functioning. As a further examination of this potential redundancy, we attempted, but failed to sufficiently knockdown Actinin-1 in KD fibroblasts by siRNA; this was probably due to the long half life of actinin (more than 30 hrs) and the low transfection efficiency of primary fibroblasts. Thus, this question of isoform functionality remains for future investigations.

In conclusion, our findings show that the α-actinin-4 is essential for maintaining the normal cell morphology, cellular and nuclear cross-sectional area, focal adhesion, contractile force and hence motility. The appropriate expression of α-actinin-4 is also important for normal proliferation of fibroblasts. Future studies should investigate why knockdown of α-actinin-4 affects the expression of myosin II and myosin light chain 2 in cancer cell and fibroblasts.
